# A post-surgical case of rib osteomyelitis and pleural abscess caused by *mycobacterium abscessus* after 6 years of lung cancer surgery

**DOI:** 10.1093/omcr/omaf212

**Published:** 2025-10-29

**Authors:** Yoshikazu Mutoh, Tomonori Sato, Yuko Oya, Takumi Umemura, Tatsuya Hioki, Yasuhiko Yamano, Kensuke Kataoka, Toshiaki Matsuda, Tomoki Kimura, Toshihiko Ichihara, Yasuhiro Kondoh

**Affiliations:** Department of Infectious Diseases, Tosei General Hospital, 160, Nishi-oiwakecho, Seto, Aichi 489-8642, Japan; Department of Respiratory Medicine and Allergy, Tosei General Hospital, 160, Nishi-oiwakecho, Seto, Aichi, 489-8642, Japan; Department of Thoracic Oncology, Aichi Cancer Center Hospital, 1-1 Kanokoden, Chikusa-ku, Nagoya, Aichi 464-8681, Japan; Department of Infection Control team, Tosei General Hospital, 160, Nishi-oiwakecho, Seto, Aichi, 489-8642, Japan; Department of Infection Control team, Tosei General Hospital, 160, Nishi-oiwakecho, Seto, Aichi, 489-8642, Japan; Department of Infection Control team, Tosei General Hospital, 160, Nishi-oiwakecho, Seto, Aichi, 489-8642, Japan; Department of Respiratory Medicine and Allergy, Tosei General Hospital, 160, Nishi-oiwakecho, Seto, Aichi, 489-8642, Japan; Department of Respiratory Medicine and Allergy, Tosei General Hospital, 160, Nishi-oiwakecho, Seto, Aichi, 489-8642, Japan; Department of Respiratory Medicine and Allergy, Tosei General Hospital, 160, Nishi-oiwakecho, Seto, Aichi, 489-8642, Japan; Department of Infectious Diseases, Tosei General Hospital, 160, Nishi-oiwakecho, Seto, Aichi 489-8642, Japan; Department of Infection Control team, Tosei General Hospital, 160, Nishi-oiwakecho, Seto, Aichi, 489-8642, Japan; Department of Respiratory Medicine and Allergy, Tosei General Hospital, 160, Nishi-oiwakecho, Seto, Aichi, 489-8642, Japan

**Keywords:** osteomyelitis, lung cancer, pleural abscess, non-tuberculosis mycobacterium (NTM), *mycobacterium abscessus*

## Abstract

A 69-year-old man followed post lung cancer surgery for 6 years presented with back pain. His imaging revealed a pleural abscess and rib osteomyelitis along with the surgical site. Therefore, bone biopsy and culture were performed and the organism was diagnosed as *Mycobacterium abscessus* by PCR method. As such, the combination antibiotics therapy initiated with imipenem, amikacin, and clarithromycin, and then continued with faropenem, minocycline, clarithromycin, and moxifloxacin for 20 months, successfully surpassing the disease without additional surgery. Although the number of non-tuberculosis mycobacterial infection has been increasing, osteomyelitis caused by *M. abscessus* is extremely rare. This time, we describe the clinical course and current treatment strategy for *M. abscessus.*

## Introduction

Recently, a great deal of Non-tuberculous mycobacteria (NTM) infection has been reported globally. Among NTM species, *M. abscessus* complex, which classified three subspecies, is considered one of the challenging diseases from the points of diagnostic methods, selection of antibiotics, and appropriate treatment duration. Here, we reported a patient who presented with post-operative rib osteomyelitis and pleural abscess caused by *M. abscessus* 6 years after lung cancer surgery. Although osteomyelitis caused by this microorganism is extremely rare, aggressive long-term combination antibiotics therapy was able to suppress the infection fortunately. Robust treatment recommendations are warranted in the future.

## Case report

**Figure 1:**
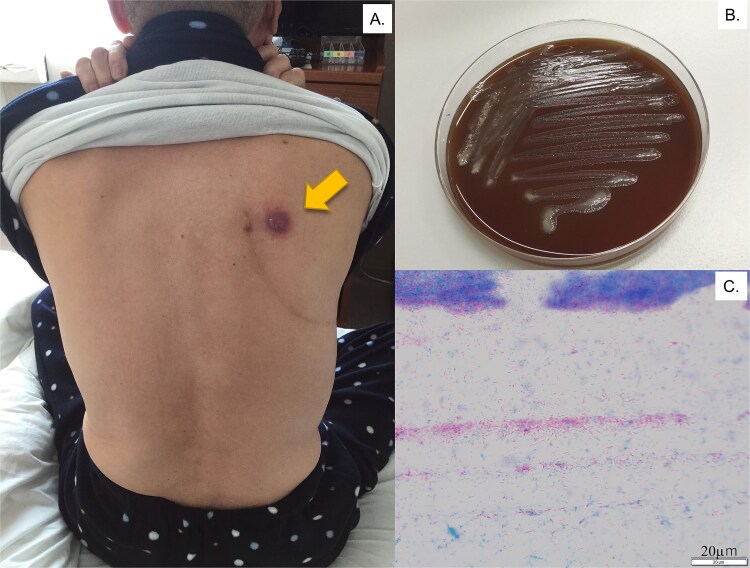
Cutaneous lesion at presentation. (A) Exudates coming out from a nodule surrounded by erythema and swelling on the patient’s back (arrow). (B) Rough and dry morphologic colonies on chocolate agar, incubating for 48 h with CO2. (C) Acid-fast bacilli were observed on Ziehl-Neelsen stain.

**Figure 2:**
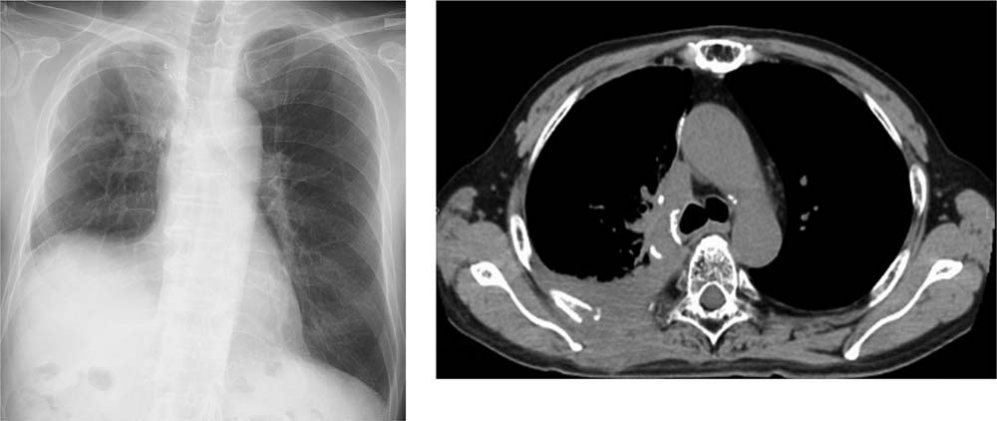
Chest X-ray and computed tomography (CT) at the initiation of treatment. CT scan reveals an osteolytic lesion and an abscess extending into the subcutaneous tissue.

A 69-year-old man was referred to the hospital with progressing right back pain and subcutaneous abscess persistent for 4 weeks (Fig. 1). The patient had also reported productive cough beginning a few days prior to consultation. Six years prior, he had a history of squamous cell carcinoma of his right upper lung with the stage of IIIA (cT3N1M0), then he subsequently received four cycles of chemotherapy with carboplatin and paclitaxel, along with concurrent radiation therapy (40 Gy). Thereafter, a right upper lobectomy via the 4th intercostal space, upper mediastinal lymph node dissection, and pulmonary angioplasty were performed. There has been no history of trauma or immunosuppressive therapy since the surgery. On examination, his vital signs were normal, and blood test showed a white blood cell count of 6800/μL and a C-reactive protein level of 3.5 mg/dL. His T-SPOT.TB test was negative and the tumor markers were within normal range. He reported no fever and no loss of body weight. Exudates were observed from subcutaneous nodules with erythema and induration on his back (Fig. 1A). A chest X-ray revealed pleural abscess in the right lung and infiltration behind his heart. A chest computed tomography (CT) image revealed an osteolytic lesion in the right fifth/sixth rib and an abscess involving latissimus dorsi muscle and subcutaneous tissue (Fig. 2). Three days after of transbronchial lung and bone biopsy and abscess culture from his back, acid-fast bacilli was found using Ziehl–Neelsen stain (Fig. 1B and C). The isolated bacteria were identified as *M. abscessus* (*M. abscessus*) complex using a DNA–DNA hybridization, and subsequently classified as *M. abscessus subsp. abscessus* or *M. abscessus subsp. Bolletii,* using a multiplex polymerase chain reaction assay detecting presence of *erm* (41) gene [1]. The minimal inhibitory concentration was evaluated by brothMIC NTM method, and the results (day3) was showed in Table 1. As such, the patient was hospitalized, then a 4-week course of intravenous imipenem, amikacin, and oral clarithromycin combination therapy was administered based on the guidelines [2]. Surgery was not performed because substantial postoperative adhesions were identified. Therefore, faropenem, minocycline, and clarithromycin, along with intravenous amikacin, were initiated and then replaced with moxifloxacin. Finally, 20 months of oral combination antibiotic treatment completed and as of 24 months after treatment completion, his symptom was completely resolved, and laboratory data showed no findings suggestive of recurrence.

**Table 1:** The minimal inhibitory concentrations of isolated *M. Abscessus* subsp. *Bolletii*, and the CLSI breakpoints for the rapidly growing mycobacteria [10]

Agent	MIC(μg/mL) of our case	MIC (μg/mL) for category:		
		Susceptible	Intermediate	Resistant
streptomycin	64			
ethambutol	64			
kanamycin	8			
rifampicin	32			
rifabutin	4			
levofloxacin	8	≤ 1	2	≥ 4
clarithromycin	> 32	≤ 2	4	≥ 8
ethionamide	> 16			
amikacin	8	≤ 16	32	≥ 64

## Discussion

We experienced a successful treated case of osteomyelitis and pleural abscess caused by *M. abscessus,* one of the species of rapidly growing mycobacterium (RGM), usually generate colonies on solid culture media within 7–14 days [3]. In a previous article reporting RGM surgical wound infection, all sixteen cases developed symptoms and had pathogens isolated within 3 weeks after surgery [4]. Additionally, the most probable causes of surgical site infection due to NTM are exposure to contaminated equipment or water during surgery. In contrast, our case presented clinical progression and yielded *M. abscessus* from the surgical site after 6 years of lung surgery. Surgical site infection caused by NTM should be considered in the differential diagnosis of patients suspected of cancer recurrence, even several years after surgery.

*M. abscessus* is classified into three distinct subspecies: *M. abscessus* subsp. *abscessus，M. abscessus* subsp. *massiliense,* and *M. abscessus* subsp. *bolletii* [5]. *M. abscessus* subsp. *bolletii* is considered to be responsible for less than 5% of *M. abscessus* complex infections [6]. In our case, osteomyelitis caused by *M. abscessus*, especially by *M. abscessus subsp. abscessus* or *M. abscessus subsp. bolletii*, is extremely rare.

The treatment of *M. abscessus* infection is not well established. The current guidelines for NTM pulmonary disease recommend more than one month of initial treatment including at least three active drugs (including azithromycin), followed by at least two to three active drugs, typically consisting of inhaled amikacin, azithromycin or clarithromycin, and oral antibiotics (such as clofazimine, linezolid, minocycline, moxifloxacin, and co-trimoxazole), as guided by antimicrobial susceptibility during continuation therapy [1, 7]. In our case, susceptibility testing was outsourced; therefore, we were not able to evaluate clarithromycin susceptibility after 14 days of incubation. However, the result at 3 days of incubation showed resistance.

In this case, although the microorganism was resistant to clarithromycin, intravenous amikacin, imipenem, and clarithromycin were initially selected in accordance with guidelines. Subsequently, we chose faropenem, minocycline and moxifloxacin. Faropenem, one of the oral β-lactam agent, has been expected to show efficacy against *Mycobacterium tuberculosis*, and previous reports have shown favorable outcome in the patient with *M. abscessus* pulmonary infection [8]. In addition, although minocycline, a tetracycline agent, has recently been reported to be inactive and lacking synergic effects with other antibiotics against *M. abscessus* [9], we selected it because this had been considered a potential oral treatment option in previous guidelines. As a result, we achieved sputum culture conversion, and the treatment was completed.

In conclusion, we experienced a case of rib osteomyelitis and pleural abscess caused by *M. abscessus.* Even several years after surgery, the pathogen may be isolated and misdiagnosed as recurrence of lung cancer. Moreover, since the treatment of *M. abscessus* remains controversial, the development of robust treatment recommendations is warranted.
